# Editorial to “Performance of the novel ANTWERP score in predicting heart function improvement after atrial fibrillation ablation in Asian patients with heart failure”

**DOI:** 10.1002/joa3.13173

**Published:** 2024-10-21

**Authors:** Satoshi Yanagisawa, Yasuya Inden, Toyoaki Murohara

**Affiliations:** ^1^ Department of Cardiology Nagoya University Graduate School of Medicine Nagoya Japan; ^2^ Department of Advanced Cardiovascular Therapeutics Nagoya University Graduate School of Medicine Nagoya Japan

**Keywords:** ANTWERP score, atrial fibrillation, catheter ablation, heart failure, reverse remodeling

In the current issue of the *Journal of Arrhythmia*, Lin et al.^1^ retrospectively investigated the predictive value of the ANTWERP score for recovery of cardiac function after atrial fibrillation (AF) ablation in 84 patients of Asian ethnicity with heart failure (HF) at a single center in Taiwan. Echocardiographic findings indicated that 68 (81%) patients had an endpoint of left ventricular ejection fraction (LVEF) recovery during an average of 8 months of follow‐up. Multivariate analysis indicated that an ANTWERP score ≤2 was an independent predictor of favorable ventricular remodeling postablation.

Indications for catheter ablation in patients with AF are expanding with technological advancement and device development. The latest European Society of Cardiology guidelines recommend a class I indication for catheter ablation in patients with AF and HF with reduced ejection fraction, possibly owing to tachycardia‐induced cardiomyopathy.^2^ Cardiac function often recovers after elimination or reduction of AF burden following catheter ablation; however, the extent of recovery varies case by case, which can affect the prognosis for HF hospitalization and mortality thereafter.

Various parameters and factors, including the etiology of HF, AF pattern, absence/presence of recurrence, baseline LVEF, onset of AF, and HF timing, are predictors of LVEF recovery and prognosis post‐AF ablation. Cardiac magnetic resonance imaging is also helpful in identifying underlying fibrosis associated with the recovery of cardiac function after catheter ablation in patients with reduced LVEF and suspected idiopathic HF. The ANTWERP score, comprising four clinical baseline parameters involving calculated score points (known etiology [2 points], QRS duration >120 ms [2 points], paroxysmal AF [1 point], and left atrial volume index [LAVI] >50 mL/m^2^ [1 point]), was first proposed in a single‐center retrospective analysis of the ANTWOORD study involving 111 patients with AF and HF with impaired LVEF (<50%) who underwent ablation at Antwerp University Hospital.^3^ The score was subsequently validated in a multicenter retrospective study involving eight European centers and 605 patients with HF, demonstrating accurate discrimination for LVEF recovery in 93% of patients with a score <2 and with LVEF recovery in only 24% of patients with a score >3.^4^ Responders were defined as those whose LVEF improved ≥50% from a baseline LVEF of 40%–50% or those with a ≥10% LVEF increase and an LVEF >40% from a baseline LVEF of <40%, exhibiting a favorable prognosis for HF hospitalization and mortality compared with nonresponders.

The current study by Lin et al.^1^ highlights the significant benefits of using the ANTWERP score for an Asian population with HF, similar to the original European study. The responder rate of 87.8% in patients with a score ≤2 was almost consistent with that of 90% in the European study. This score is simple to calculate, no specific invasive assessments are required, and all parameters involve a plausible pathology for recovery of cardiac function. However, caution is needed when applying the same cutoff value of the score, particularly for a QRS duration of 120 ms and an LAVI of 50 mL/m^2^ for patients of Asian ethnicity with HF. For example, only 14.3% of patients in the current study had a QRS duration >120 ms, which was lower than the 29.8% reported in the original European study. The QRS duration in populations of Asian ethnicity is generally shorter than that in populations of European ethnicity. This difference may be because of the smaller body size among Asian populations.^5^ The average QRS duration in a healthy community‐based cohort of Chinese, Malay, and Indian individuals was 89 ms in males and 83 ms in females, whereas the average QRS duration in a healthy Caucasian cohort in the Framingham heart study was 97 and 87 ms in males and females, respectively.^5^ Determining an original cutoff value specific to patients of Asian ethnicity with HF (e.g., 100 ms) would be useful to differentiate the prognosis more accurately,^5^ given the original cutoff values concerning QRS duration and LAVI in the ANTWOORD study were subjectively defined without apparent statistical analysis.^3^ Moreover, the lower prevalence of known etiology (28.6% vs. 40.2%, respectively) and higher paroxysmal AF (65.5% vs. 20.2%, respectively) reported in Lin et al.'s study^1^ may represent a distinct data set of the baseline characteristics compared with the European study, possibly causing the different response rates and outcomes when adjusting for baseline etiology. Specifically, focused cardiac magnetic resonance imaging to determine the underlying etiology is likely to be critical to enhance the rate of known HF etiology associated with poor LVEF recovery and to increase the score's predictive value accuracy. Given that the etiology of HF is the only manageable factor among the four ANTWERP parameters, the results underscore the importance of physicians making every effort to evaluate the underlying HF disease in detail and to stratify the prognosis thereafter, despite there being some differences in HF etiology between European and Asian populations. Moreover, social and economic barriers may limit examinations in some Asian countries.

The optimal timing for LVEF recovery post‐AF ablation remains unclear. Most studies have focused on reverse remodeling after 6–12 months of ablation; however, the evidence suggests that LVEF recovery continues up to 2 years postprocedure, which raises a concern that excessive early echocardiographic evaluation may underestimate further LVEF recovery and reverse remodeling. Nonetheless, early assessment of changes in cardiac function is beneficial to recognize responses to catheter ablation for eliminating AF at an early stage and can facilitate the prompt management of HF and the adding/titration of cardioprotective drugs, which may improve the prognosis in patients with insufficient response. In this regard, noninvasive assessment using the ANTWERP score is helpful in determining treatment planning and decision‐making for patients with HF prior to AF ablation. A further randomized large sample‐sized study focusing on patients of Asian ethnicity with HF is needed to evaluate the ANTWERP score and to determine an optimal cutoff value for such patients.

## CONFLICT OF INTEREST STATEMENT

Dr. Yanagisawa is affiliated with a department sponsored by Medtronic, Japan. Other authors have no conflicts of interest to declare.
